# Brain-computer interface for post-stroke upper-limb recovery: a systematic review integrating clinical efficacy with neuroplasticity evidence

**DOI:** 10.3389/fneur.2026.1895484

**Published:** 2026-09-03

**Authors:** Yu Wu, Feilong Zhu, Qiumeng Dong, Ming Zhang

**Affiliations:** 1 Department of Sports Science, Zhejiang University, Hangzhou, China; 2 Physical Education and Sports Science Department, National Institute of Education, Nanyang Technological University, Singapore, Singapore; 3 School of Sports Medicine and Rehabilitation, Beijing Sport University, Beijing, China; 4 School of Rehabilitation Medicine, Nanjing Medical University, Nanjing, China; 5 Department of Physical Therapy, The Affiliated Xuzhou Rehabilitation Hospital of Xuzhou Medical University, Xuzhou, China

**Keywords:** brain-computer interface, meta-analysis, neuroplasticity, stroke, upper limb

## Abstract

**Objective:**

Over 20 systematic reviews have evaluated brain-computer interface (BCI) training for post-stroke upper-limb rehabilitation, but published syntheses have focused predominantly on clinical efficacy, and the neuroplasticity mechanisms remain comparatively underexplored. This review integrates pooled clinical efficacy with a structured synthesis of neuroplasticity evidence from the same trials.

**Methods:**

PubMed, Embase, CINAHL via EBSCO, Web of Science, Scopus, and Cochrane CENTRAL were searched from inception to May 2026. Randomized controlled trials (RCTs) of BCI-based training for post-stroke upper-limb recovery were eligible. Two parallel syntheses were conducted. A random-effects meta-analysis pooled mean differences for three upper-limb motor outcomes, RoB 2 risk-of-bias assessment, GRADE certainty-of-evidence grading, and publication-bias diagnostics. Neuroplasticity evidence was synthesized qualitatively following the Synthesis Without Meta-analysis (SWiM) guideline.

**Results:**

Thirty-five RCTs (*n* = 1,188) were included. BCI training improved Fugl-Meyer Assessment-Upper Extremity [FMA-UE; MD = 4.55, 95% CI (2.84, 6.25), *P* < 0.001], Action Research Arm Test [ARAT; MD = 3.90, 95% CI (1.31, 6.50), *P* = 0.003], and Wolf Motor Function Test [WMFT; MD = 8.53, 95% CI (3.01, 14.04), *P* = 0.002]. Comparator type significantly moderated the effect [*Q*-between(3) = 13.43, *P* < 0.01], ranging from MD = 6.70 against usual care to MD = 1.97 against sham-contingent controls; stroke stage, signal strategy, and feedback modality did not. Qualitative synthesis of 22 trials identified multi-level neuroplastic changes spanning corticospinal excitability, sensorimotor rhythm modulation, and network reorganization. A subset of studies reported correlations between neuroplastic changes and motor improvement.

**Conclusion:**

This systematic review found that BCI-based multimodal rehabilitation improves post-stroke upper-limb motor function, with the estimated effect varying by comparator type. Neuroplasticity outcomes were examined across electrophysiological, hemodynamic, and structural modalities, but the current evidence is too heterogeneous and sparse to support a mechanistic account of recovery. Further research is needed to determine how training-associated neural changes relate to functional improvement.

**Systematic review registration:**

https://www.crd.york.ac.uk/prospero/view/CRD420251053630, identifier: CRD420251053630.

## Introduction

The modern paradigm of stroke rehabilitation has shifted from compensatory training toward activity-dependent neuroplasticity as the primary driver of functional recovery (1, 2). This principle is especially consequential for the upper limb, where restoration of hand and arm function is a primary determinant of independence and quality of life (3). Despite sustained clinical effort, persistent upper-limb impairment affects more than half of stroke survivors, many of whom experience chronic deficits that constrain essential daily activities (4, 5). A core contributor, particularly in those with severe motor impairment, is the inability to generate sufficient voluntary movement to engage meaningfully in intensive, task-specific training (6). This creates a self-reinforcing cycle: reduced movement limits task-relevant neural activation, which in turn deprives the sensorimotor cortex of the input required to sustain plasticity-dependent recovery (7). Interrupting this cycle requires technologies that can engage residual cortical motor circuits even without overt movement.

Brain-computer interfaces (BCIs) address this gap directly (8). By decoding cortical signals associated with motor intention and delivering contingent sensory feedback in real time, BCIs form an artificial closed-loop sensorimotor circuit that bypasses damaged pathways (9). The therapeutic rationale is that contingent feedback enables patients to volitionally modulate cortical activity that is otherwise inaccessible during attempted movement (10). This architecture captures residual cortical activity accompanying motor intention as a trainable signal when overt movement is not possible, such that practice-dependent plasticity is driven by motor intention rather than by physical execution.

Numerous systematic reviews and meta-analyses have evaluated BCI training for post-stroke upper-limb rehabilitation. These syntheses have primarily examined whether BCI training improves motor outcomes and which protocol characteristics are associated with larger effects. Several reviews have also addressed moderators or individual neural measures. These syntheses, however, have focused predominantly on clinical efficacy, with the neuroplasticity mechanisms through which BCI training may facilitate recovery remaining comparatively underexplored. Moreover, the cognitive processes that sustain the BCI feedback loop are not captured by standard motor outcome measures and have therefore remained invisible to efficacy-focused meta-analyses. Without a mechanistic foundation, patient selection has no principled neural basis, protocol adaptation lacks guidance from individual response markers, and BCI cannot advance from an effective group-level treatment to a tool for individualized neurorehabilitation.

The present review was designed to address this gap through two complementary analyses applied to the same set of randomized trials. A meta-analysis of upper-limb motor outcomes quantifies the magnitude and consistency of BCI-driven clinical benefit. A systematic qualitative synthesis examines the neuroplasticity evidence reported across those same trials. Examining both dimensions within a single evidence base allows the review to assess whether the treatment effect is accompanied by neural reorganization, and to identify which patterns recur across studies.

## Methods

This systematic review and meta-analysis was reported in accordance with the Preferred Reporting Items for Systematic Reviews and Meta-Analyses (PRISMA) guidelines (11) (Supplementary Table S1). The study protocol was registered on PROSPERO (CRD420251053630). Although the original protocol included cognitive function as a secondary outcome, this was excluded from the final quantitative analysis because too few included RCTs reported standardized cognitive data. The focus of this review was therefore restricted to upper-limb motor function and neuroplasticity.

### Search strategy and eligibility criteria

A systematic literature search was performed in the following databases from their inception to May 2026: PubMed, Embase, CINAHL via EBSCO, Web of Science, Scopus and the Cochrane Central Register of Controlled Trials (CENTRAL). The search strategy was structured around key concepts of the patient population (stroke) and the intervention (BCI). For each concept, a combination of Medical Subject Headings (MeSH) and a comprehensive set of free-text keywords searched in the title and abstract ([Title/Abstract]) was used to maximize sensitivity. The core search logic in PubMed involved combining terms such as (“Brain-Computer Interfaces”[Mesh] OR “Brain-Machine Interface”[Title/Abstract] OR “Neural Interface Technology”[Title/Abstract]) with terms like (“stroke”[Mesh] OR “Cerebrovascular Accident”[Title/Abstract] OR “CVA”). The full, unabridged search query as executed in PubMed is provided in the Supplementary Table S2, along with the adapted strategies for other databases. Additionally, we manually screened the reference lists of included studies and relevant review articles to identify any potential additional publications. No search of gray literature sources, including trial registries and conference proceedings, was conducted.

Studies were included if they met the following criteria: (1) population: adult patients diagnosed with stroke (ischemic or hemorrhagic); (2) intervention: BCI-based training paradigm targeting upper-limb rehabilitation; (3) comparison: a control group receiving no intervention, sham intervention, or another form of conventional therapy; (4) participants were required to have sufficient cognitive capacity to engage with the BCI task, as determined by each trial's own screening procedure; (5) outcomes: at least one standardized upper-limb function measure was reported at the primary post-intervention assessment point, with sufficient data to calculate an effect size; follow-up data were extracted where available but analyzed separately; and (6) publication type/language: peer-reviewed full-text articles published in English.

### Study selection and data extraction

Two reviewers (YW and FZ) independently screened the titles and abstracts of the retrieved records. Full texts of potentially eligible articles were then obtained and assessed for final inclusion. Any disagreements were resolved through discussion or by consulting a third reviewer (MZ).

A standardized data extraction form was used to collect information from each included study. The extracted data included: (1) basic study information (first author, year of publication, study ID); (2) participant characteristics (sample size, stroke stage, mean age, mean duration since stroke, lesion type and location); (3) BCI intervention details (signal type, output type, total sessions, total weeks, sessions per week); (4) control intervention details; and (5) outcome measures, including mean, standard deviation (SD), and sample size for both groups at pre-intervention, post-intervention, and all available follow-up time points. Definitions, score ranges, and scoring directions for the three pooled outcomes are provided in Supplementary Table S4. For studies reporting neurological outcomes, we also extracted several key details: the measurement tool [electroencephalography (EEG), functional magnetic resonance imaging (fMRI), the type of measure (activation, connectivity), and the reported findings (*P*-values, correlation coefficients)]. For studies that did not report outcome data in the standard mean and SD format, we applied systematic conversion and estimation methods. The specific procedures for handling data presented in graphical form, as change-from-baseline scores, or as median and interquartile range are detailed in Table 1. These converted or graphically extracted values were retained in the primary analyses. Data extraction was performed by one reviewer (YW) and cross-checked by a second reviewer (MZ) to ensure accuracy.

**Table 1:** Methods for handling non-standard outcome data.

Data reporting format	Problem	Solution and rationale
Graphical Data	Mean/SD presented in figures (e.g., bar charts)	Values extracted using WebPlotDigitizer (v4.7, Ankit Rohatgi, Pacifica, Canada). SE converted to SD (SD = SE × √n).
Change-from-Baseline	Only baseline mean/SD and mean change reported	Post-intervention mean calculated (M_post = M_pre + M_change). Missing post-intervention SD imputed with baseline SD (a conservative approach per Cochrane Handbook (12)).
Median & Quartiles	Data reported as Median and Interquartile Range (IQR) or Quartiles (Q1, Q3)	Mean estimated as approximately equal to the median. SD estimated using the formula: SD ≈ IQR/1.35 (12, 13)

SD, Standard Deviation; SE, Standard Error; n, Sample Size; M_post, Mean post-intervention; M_pre, Mean pre-intervention; M_change, Mean of the change scores; IQR, Interquartile Range; Q1, Q3, First Quartile and Third Quartile.

### Study risk of bias assessment and quality of evidence

The risk of bias for the included RCTs was assessed using the RoB 2 (14), which evaluates five domains: randomization process, deviations from intended interventions, missing outcome data, measurement of the outcome, and selection of the reported result. An overall risk of bias judgment (low risk, some concerns, or high risk) was assigned to each study. Two reviewers (YW and FZ) independently performed the quality assessment, with discrepancies resolved by consensus.

The certainty of the evidence for each outcome was assessed using the Grading of Recommendations Assessment, Development and Evaluation (GRADE) approach (15), which evaluates five domains: risk of bias, inconsistency, indirectness, imprecision, and publication bias. The certainty of evidence was graded as high, moderate, low, or very low. A summary of findings table was generated using GRADEpro software (McMaster University and Evidence Prime, Hamilton, ON, Canada) to present the pooled effects and their corresponding certainty levels.

### Qualitative synthesis of neuroplastic mechanisms

Because neurophysiological measures are not directly comparable across modalities (for example, EEG event-related desynchronization, fMRI functional connectivity, and TMS motor evoked potentials index different aspects of neural function on different scales), quantitative pooling of neuroplasticity findings was not appropriate. Accordingly, neuroplasticity findings were synthesized qualitatively with reference to the Synthesis Without Meta-analysis (SWiM) reporting guideline (16). Studies were grouped by neurophysiological modality (electrophysiological, hemodynamic or structural, and brain-behavior correlation), which served as the basis for structuring the synthesis. Within each group, we recorded the direction of each reported effect and assessed the consistency of findings by examining whether independent studies pointed in the same direction. Because the included measures were reported on non-commensurable scales, we did not perform vote-counting based on statistical significance alone. The certainty and weight given to each finding were informed by the risk-of-bias assessment of the contributing study, such that findings from studies at high risk of bias were interpreted with greater caution.

### Statistical analysis

All statistical analyses were performed using R (version 4.5.1; R Foundation for Statistical Computing, Vienna, Austria). Statistical significance was set at *P* < 0.05. For clinical outcomes measured on the same scale across studies, effect sizes were expressed as mean differences (MDs) with 95% confidence intervals (CIs). Random-effects models were applied because between-study heterogeneity was anticipated in participant characteristics and intervention protocols. The DerSimonian–Laird estimator was used for the primary outcome analyses, while restricted maximum likelihood was used for subgroup analyses where estimable. Dose-response relationships between training dosage and Fugl-Meyer Assessment-Upper Extremity (FMA-UE) improvement were explored in an exploratory meta-regression, with the full model specification provided in the Supplementary methods.

The included BCI interventions were characterized along two principal dimensions: the signal-acquisition paradigm driving the interface and the feedback modality delivering contingent stimulation. To explore sources of heterogeneity in FMA-UE improvement, we examined these two intervention dimensions together with three contextual factors (stroke stage, comparator type, and study design) in five subgroup analyses: (1) stroke stage [subacute (1 week−6 months), chronic (> 6 months), and mixed populations (“mix-early”: acute and subacute patients < 6 months post-stroke; “mix-late”: subacute and chronic patients spanning the 6-month threshold)] (17); (2) BCI signal-acquisition paradigm [motor imagery (MI), motor attempt (MA), action observation (AO), steady-state visual evoked potential (SSVEP), combined MI and AO (MI_AO), or studies with unspecified paradigms]; (3) feedback modality [robotic or exoskeletal devices, orthoses and soft robotic gloves, functional electrical stimulation (FES), or visual and haptic feedback]; (4) comparator type [sham or neural-contingency controls (feedback not contingent on the patient's own neural activity), active dose-matched rehabilitation (an alternative therapy delivered at matched intensity), device-assisted non-BCI controls (the same peripheral device without BCI control), or usual care and conventional rehabilitation]; and (5) study design [add-on trials (BCI added to a background therapy given to both arms) vs. comparative trials (BCI tested against a different therapy)]. Comparator type and study design were examined as exploratory analyses.

Heterogeneity was quantified using Cochran's Q test, the *I*^2^ statistic, and the between-study variance estimator τ^2^. *I*^2^ values exceeding 50% were considered indicative of substantial heterogeneity (12, 18). To assess the robustness of the pooled estimates to data-handling decisions, we performed sensitivity analyses restricted to studies that reported means and standard deviations directly, excluding values obtained by graphical extraction, SD imputation, or conversion from medians and interquartile ranges. Publication bias for outcomes with a sufficient number of studies was assessed visually using funnel plots and statistically using Egger's linear regression test (18). When asymmetry was suggested, trim-and-fill analysis was used as an exploratory sensitivity analysis to examine whether the overall interpretation materially changed after accounting for potentially missing small studies.

## Results

### Study selection and characteristics

**Figure 1:**
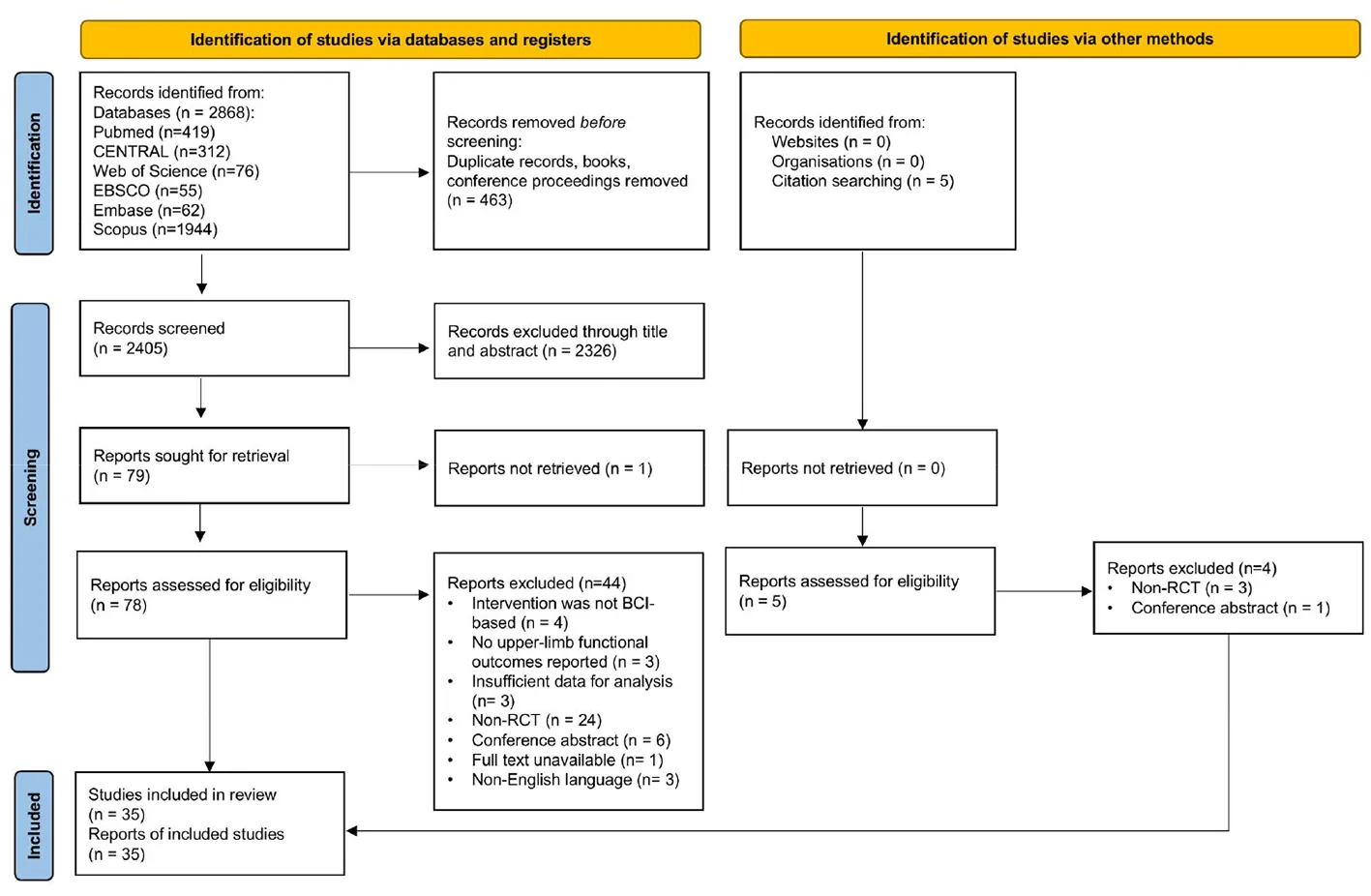
PRISMA flow diagram of study selection.

A total of 35 randomized controlled trials were included in this review (Figure 1). The main characteristics of these studies are summarized in Table 2. The studies, published between 2013 and 2025, collectively enrolled 1,188 participants with sample sizes ranging from 11 to 128. Most studies evaluated motor-imagery-based BCI paradigms combined with robotic, orthotic, visual, or electrical feedback, whereas control conditions included sham BCI, conventional rehabilitation, or device-based training without contingent neural control.

**Table 2:** Characteristics of included randomized controlled trials.

References	N (Exp/Con)	Age (yr), mean (SD) (Exp/Con)	Stroke stage	Duration mean (SD) (months)	BCI intervention	Control intervention	Dosage	Outcome measure
Zhang et al. (19)	15/15	55.27 (9.93)/62.60 (10.99)	acute/subacute	12.40 (4.26)/12.00 (3.36) (days)	MI-BCI + Exoskeleton	CR	30 min, 5/wk, 4 wks	FMA-UE, MBI, fNIRS
Lu et al. (20)	20/19	52.60 (14.48)/50.42 (13.61)	chronic	12.00 (6.90)/9.21 (7.20)	CR + MI-BCI + Visual + Tactile Robot	CR+MI	30 min, 5/wk, 4 wks	FMA-UE, ARAT, sEMG, fMRI
Cantillo-Negrete et al. (21)	10/9	47.18 (15.74)/55.78 (14.96)	chronic	12.67 (5.79)/10.33 (6.26)	Physiotherapy + MI-BCI + Hand Orthosis	Random Orthosis Movement	NR, 5/wk, 6 wks	FMA-UE, ARAT, DTI, TMS
Kim et al. (22)	12/13	49.0 (16.9)/46.0 (12.8)	chronic	18.2 (16.2)/22.2 (23.4)	MI-BCI	Sham-BCI	30 min, 5/wk, 4 wks	MRC-WE, AROM-WE, FMA-UE, EEG
He et al. (23)	25/23	58.6 (12.8)/58.3 (12.8)	acute/subacute	33.3 (18.5)/41.8 (19.1) (days)	MI/MA-BCI + VR + Pedal Robot	Sham BCI	20 min, 5/wk, 2 wks	FMA-UE
Ji et al. (24)	20/19	61.75 (10.35)/60.05 (14.35)	subacute	49.50 (23.36)/42.42 (24.50) (days)	CR+BCI-SRG	CR+SRG	20 min, 5/wk, 4 wks	ARAT, FMA-UE. MBI
Zhang et al. (25)	14/14	54.9 (8.7)/60.8 (11.5)	subacute	1.25 (0.88)/1.29 (0.42)	CR + MI-BCI + Robot + tDCS	CR + tDCS	20 min, 5/wk, 4 wk	FMA-UE ARAT Timing: 0, 4 wk
Brunner et al. (26)	15/20	56.0 (14.0)/57.0 (11.0)	subacute	1.1 (0.5)/1.0 (0.3)	MI-BCI+ Visual Feedback	CR	NR, 3–4/wk, 3–4 wk	ARAT Timing:0, 12 wk after stroke
Krueger et al. (27)	6/6	NR	acute/subacute	NR	MA-BCI + FES	Sham-Random-FES	NR, 5/wk, 3–5 wk	FMA-UE Timing: 0, 3–5 wk
Zanona et al. (28)	23/21	62.2 (9.8)/61.0 (3.0)	subacute/chronic	13.9 (6.0)/12.5 (6.7)	CR + MI-BCI + Mental Practice	CR (time-matched)	NR, 5/wk, 2 wk	FMA-UE Timing:0, 2 wk
Wang et al. (29)	64/64	NR	subacute	NR	CR + MI-BCI + Robot + Mindfulness	CR	20 min, 5/wk, 4 wk	FMA-UE Timing: 0, 4 wk
Liu et al. (30)	30/30	52.5/53.0	subacute	18.5 (NR)/18.0 (NR)	CR + MI-BCI + Visual Feedback	CR	20 min, 5/wk, 3 wk	FMA-UE WMFT Timing: 0, 3 wk
Fu et al. (31)	30/31	55.9 (11.1)/59.0 (14.5)	subacute	2.6 (4.3)/2.1 (5.0)	CR + MI-BCI + Exoskeleton	CR	30 min, 5/wk, 4 wk	FMA-UE Timing: 0, 4 wk
Liao et al. (32)	8/9	61.5 (3.8)/61.0 (3.7)	subacute	20.1 (2.2)/20.6 (2.4) (days)	CR + MI-BCI + Visual Feedback	CR	NR, 5/wk, 3 wk	FMA-UE Timing: 0, 3 wk
Guo et al. (33)	10/10	60.2 (9.3)/56.9 (6.1)	chronic	11.7 (5.4)/10.9 (7.9)	CR + SSVEP-BCI + Robot	CR	20 min, 5/wk, 2 wk	FMA-UE WMFT Timing: 0, 2 wk
Li et al. (34)	12/12	43.8 (14.7)/55.0 (12.2)	subacute/chronic	4.3 (2.6)/4.0 (12.6)	CR + AO-BCI + Visual Feedback	CR	40 min, 5/wk, 2 wk	FMA-UE WMFT Timing: 0, 2 wk
Ma et al. (35)	20/20	50.9 (12.6)/58.3 (11.2)	chronic	5.9 (3.0)/6.5 (3.4)	CR + MI-BCI + Exoskeleton	CR	40 min, 5/wk, 2 wk	FMA-UE Timing: 0, 2 wk
Zhan et al. (36)	12/12	NR	chronic	40.1 (40.1)/51.6 (51.1)	CR + MI-BCI + FES	CR + FES	40 min, 5/wk, 4 wk	FMA-UE Timing: 0, 4 wk
Hu et al. (37)	7/5	44.9 (7.5)/60.4 (16.8)	subacute/chronic	7.9 (6.5)/7.3 (4.5)	MI-BCI + Visual Feedback	Classic MI	NR	FMA-UE Timing: NR
Chen et al. (38)	7/7	41.6 (12.0)/52.0 (11.1)	subacute/chronic	3.1 (1.7)/3.9 (1.5)	MA-BCI + Haptic Feedback	CR	3/wk, 4 wk	FMA-UE Timing: 0, 4 wk
Cheng et al. (39)	5/6	62.4 (4.7)/61.4 (4.5)	subacute/chronic	15.7 (9.9)/29.2 (8.5)	CR + MI-BCI + Soft Robotic Glove	CR + SRG	17 min, 3/wk, 6 wk	FMA-UE ARAT Timing: 0, 6 wk
Miao et al. (40)	8/8	48.8 (15.6)/50.3 (16.8)	chronic	18.3 (11.6)/11.1 (4.9)	CR + FES + MI-BCI	CR	NR, 3/wk, 3 wk	FMA-UE Timing: 0, 4 wk
Wu et al. (41)	24/22	62.9 (10.6)/64.8 (7.2)	subacute	2.1 (0.3)/2.0 (NR)	CR + MI-BCI + Visual Feedback	CR	NR, 5/wk, 4 wk	FMA-UE Timing:0, 4 wk
Biasiucci et al. (42)	14/13	55.5 (10.0)/58.9 (12.1)	chronic	40.2 (46.6)/32.2 (30.8)	CR + MI-BCI + FES	CR + Sham-FES	60 min, 2/wk, 5 wk	FMA-UE Timing: pre, post, and follow-up
Frolov et al. (43)	55/19	58.0 (23.0)/58.0 (20.3)	subacute/chronic	8.0 (12.2)/8.0 (16.2)	MI-BCI + Exoskeleton	Sham-BCI	NR	FMA-UE Timing: before and after intervention
Kim et al. (44)	15/15	59.1 (8.1)/59.9 (9.8)	chronic	8.3 (2.0)/7.9 (1.8)	CR + AO-BCI + FES	CR	30 min, 5/wk, 4 wk	FMA-UE Timing: 0, 4 wk
Jang et al. (45)	10/10	61.1 (13.8)/61.7 (12.1)	subacute	4.4 (1.0)/4.1 (0.7)	CR + AO-BCI + FES	CR + FES	20 min, 5/wk, 6 wk	MFT Timing: 0, 6 wk
Pichiorri et al. (46)	14/14	64.1 (8.4)/59.6 (12.7)	subacute	2.7 (1.7)/2.5 (1.2)	CR + MI-BCI + Visual Feedback	CR + MI	30 min, 3/wk, 4 wk	FMA-UE Timing: 0, 4 wk
Ang et al. (47)	11/15	48.5 (13.5)/53.6 (9.5)	chronic	12.6 (9.6)/7.7 (6.0)	MI-BCI + MIT-Manus Robot	Robotics (Manus)	52 min, 3/wk, 4 wk	FMA-UE Timing: 0, 4 wk
Ang et al. (48)	7/8	54.0 (8.9)/51.1 (6.3)	chronic	9.4 (2.1)/13.1 (5.0)	CR + MI-BCI + Haptic Knob Robot	CR + HK	60 min, 3/wk, 6 wk	FMA-UE Timing:0, 6 wk
Li et al. (49)	8/7	67.0 (4.8)/67.1 (6.0)	subacute	2.7 (2.2)/2.8 (2.0)	CR + BCI + FES	CR + FES	20 min, 5/wk, 8 wk	FMA-UE Timing: 0, 8 wk
Mihara et al. (50)	10/10	56.1 (7.5)/56.2 (7.5)	subacute	4.8 (1.2)/4.1 (1.3)	MI-BCI + Visual Feedback	Sham-BCI	20 min, 3/wk, 2 wk	FMA-UE ARAT Timing: 0, 2 wk
Ramos-Murguialday et al. (51)	16/16	49.3 (12.5)/50.3 (12.2)	chronic	66.0 (45.0)/71.0 (72.0)	Physiotherapy + SMR-BCI + Orthosis	Physiotherapy + Sham-BCI	NR, 5/wk, 4 wk	FMA-UE Timing: 0, 8 wk
Lee et al. (52)	13/13	55.2 (11.6)/58.3 (9.2)	chronic	7.5 (1.6)/8.3 (2.0)	CR + FES + AO-BCI	CR + FES	30 min, 5/wk, 4 wk	FMA-UE WMFT Timing: 0, 4 wk
Chen et al. (53)	16/16	59.3/57.9	chronic	6.3 (NR)/5.7 (NR)	MI-BCI + FES	NMES	40 min, ~3.7/wk, 3 wk	FMA-UE Timing:0, 3 wk

Exp, experimental group; Con, control group; yr, year; SD, standard deviation; mo, month(s); wk, week(s); NR, not reported; BCI, brain-computer interface; MI, motor imagery; MA, motor attempt; SMR, sensorimotor rhythm; CR, conventional rehabilitation; SRG, soft robotic glove; VR, virtual reality; FES, functional electrical stimulation; tDCS, transcranial direct current stimulation; HK Robot, Haptic Knob robot; MIT-Manus Robot, Massachusetts Institute of Technology Manus robot; MRC-WE, Medical Research Council score for wrist extension; AROM-WE, active range of motion of wrist extension; FMA-UE, Fugl-Meyer Assessment-Upper Extremity; ARAT, Action Research Arm Test; WMFT, Wolf Motor Function Test; MBI, Modified Barthel Index; EEG, electroencephalography; fMRI, functional magnetic resonance imaging; fNIRS, functional near-infrared spectroscopy; DTI, diffusion tensor imaging; TMS, transcranial magnetic stimulation; sEMG, surface electromyography. Stroke duration values originally reported in days were converted to months for table standardization (30.4 days = 1 month).

### Methodological quality and risk of bias

**Figure 2:**
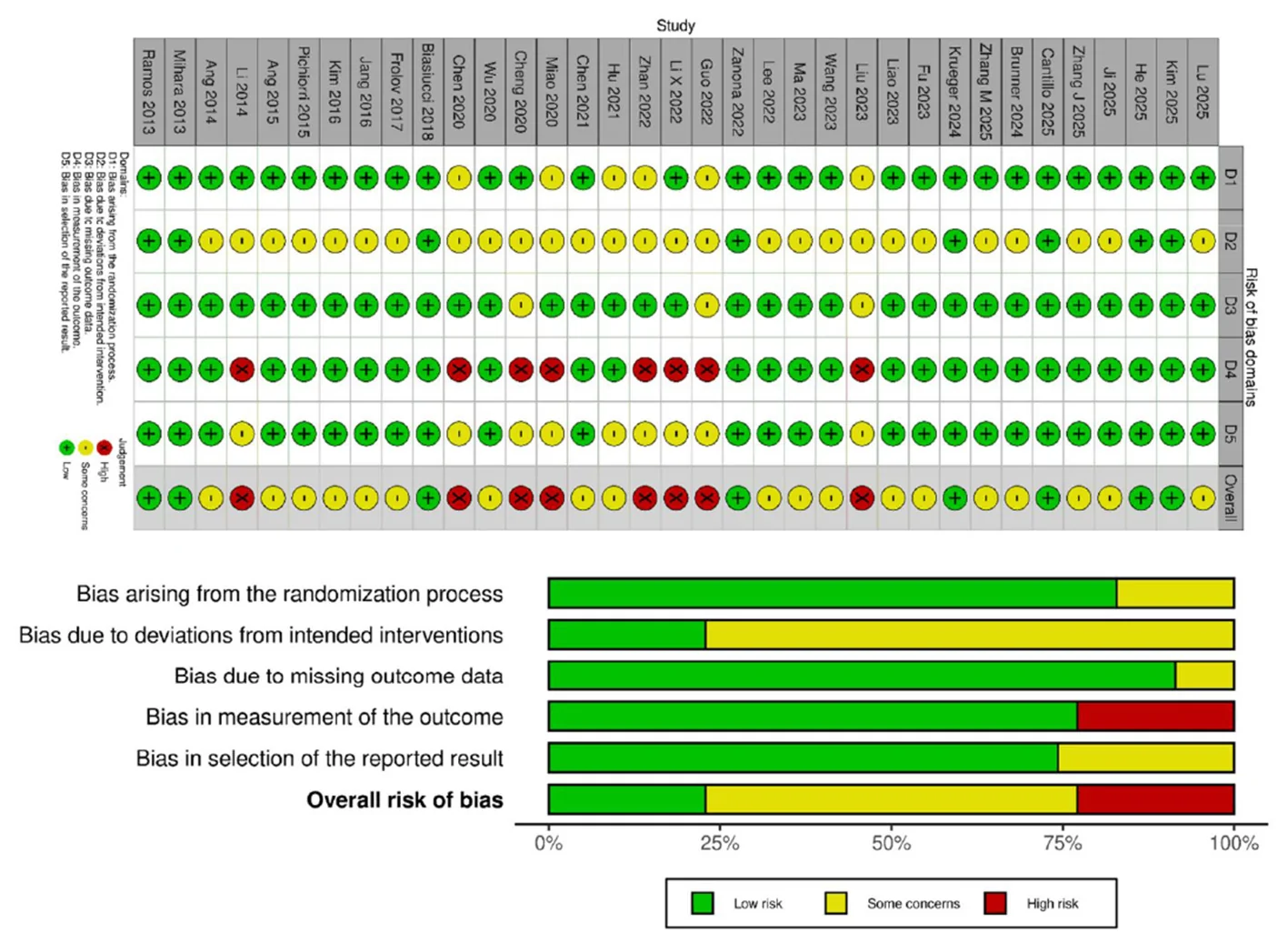
Risk of bias summary for included randomized controlled trials.

The risk of bias assessment for the 35 included studies is summarized in Figure 2. Overall, 8 studies (22.9%) were rated as low risk of bias, 19 (54.3%) as having some concerns, and 8 (22.9%) as high risk of bias. Deviations from intended interventions and outcome measurement were the most common sources of bias; randomization procedures were generally better reported. Study-level ratings for each domain are provided in Supplementary Table S3.

### Effects on upper-limb functional outcomes

We compared BCI training with control interventions on three upper-limb outcomes: the FMA-UE as the primary outcome, and the Action Research Arm Test (ARAT) and Wolf Motor Function Test (WMFT) as secondary outcomes. Across the studies contributing outcome data, 22 reported means and standard deviations directly and 12 required conversion (six from median and interquartile range, five from graphically presented data, and one by SD imputation); all five WMFT studies reported directly, so these conversions affected only the FMA-UE and ARAT analyses.

**Figure 3:**
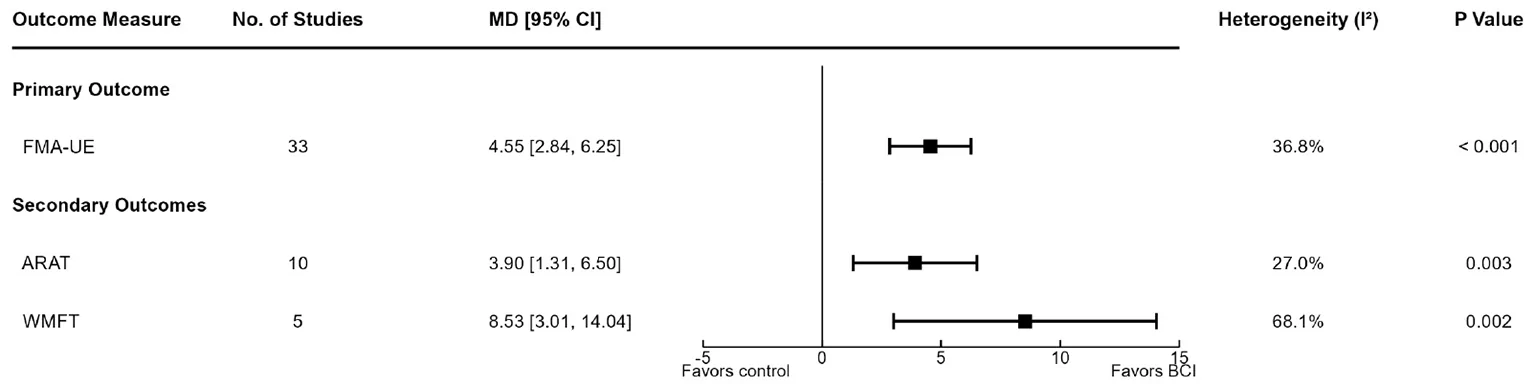
Forest plot of the effects of BCI training on upper-limb function. ARAT, Action Research Arm Test; BCI, brain-computer interface; CI, confidence interval; FMA-UE, Fugl-Meyer Assessment-Upper Extremity; MD, mean difference; WMFT, Wolf Motor Function Test.

For the FMA-UE, the primary analysis pooled 33 of the 35 included studies (*N* = 1,154; two studies were excluded because group-level data suitable for pooling were unavailable) and showed that BCI-based training improved upper-limb motor impairment compared with control interventions, with a pooled MD of 4.55 [95% CI (2.84, 6.25), *P* < 0.001]. Heterogeneity across studies was low to moderate [τ^2^ = 7.01; *I*^2^ = 36.8%; *Q*(32) = 50.62, *P* = 0.019] (Figure 3). A sensitivity analysis restricted to the 22 studies reporting mean and SD directly (excluding graphically extracted, imputed, or median-based values) yielded a comparable estimate [MD = 4.47, 95% CI (2.74, 6.21); *I*^2^ = 28.0%], indicating that data transformations did not materially affect the pooled result. The detailed forest plot for FMA-UE is shown in Supplementary Figure S1.

For the ARAT, the analysis including 10 studies (*N* = 311) showed an improvement following BCI training. The pooled MD was 3.90 [95% CI (1.31, 6.50), *P* = 0.003]. Heterogeneity was low [τ^2^ = 4.16; *I*^2^ = 27.0%; *Q*(9) = 12.32, *P* = 0.196] (Figure 3). The detailed forest plot for ARAT is shown in Supplementary Figure S2. A sensitivity analysis restricted to the five ARAT studies reporting mean and SD directly yielded a numerically larger estimate with overlapping confidence intervals [MD = 5.34, 95% CI (2.30, 8.37); *I*^2^ = 21.7%] compared with the primary analysis. Given the small number of studies in each subset, this shift should be interpreted with caution; the direction and statistical significance of the effect were preserved.

All five WMFT studies used the Functional Ability Scale (higher scores indicating better function). The pooled analysis (*N* = 176) showed a significant improvement following BCI training [95% CI (3.01, 14.04), *P* = 0.002]. Substantial heterogeneity was observed [τ^2^ = 24.14; *I*^2^ = 68.1%; *Q*(4) = 12.56, *P* = 0.014] (Figure 3). The detailed forest plot for WMFT is shown in Supplementary Figure S3.

Among the included studies, only one (Biasiucci et al., (42)) reported upper-limb outcomes at a follow-up assessment beyond the primary post-intervention time point. A follow-up meta-analysis was therefore not feasible, and follow-up data were not pooled.

### Certainty of evidence

Based on the GRADE assessment, the certainty of evidence for the FMA-UE primary outcome was graded as moderate, downgraded primarily due to risk of bias. For secondary outcomes, the certainty was graded as low for both ARAT and WMFT, downgraded due to inconsistency and imprecision. Detailed GRADE evidence profiles are presented in Supplementary Table S5.

### Moderator analyses of FMA-UE

**Figure 4:**
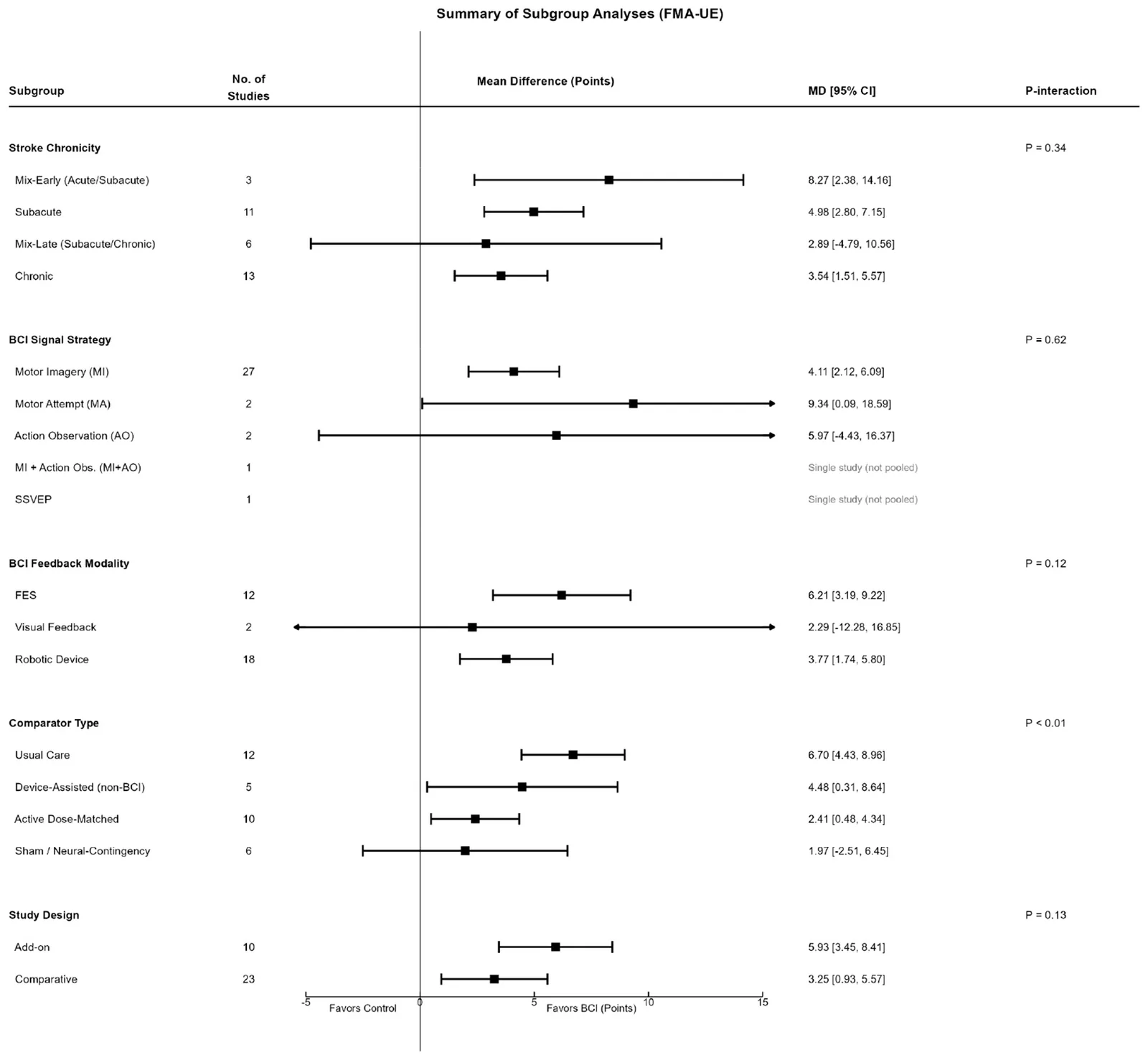
Subgroup analyses of BCI efficacy on FMA-UE scores stratified by key moderators. FMA-UE, Fugl-Meyer Assessment-Upper Extremity; MD, mean difference.

To systematically explore potential sources of heterogeneity and factors influencing BCI efficacy, we conducted a series of subgroup analyses. The comprehensive results are summarized in Figure 4. Detailed subgroup analyses are provided in Supplementary Figures S4–S8.

Subgroup analysis stratified by stroke stage identified four populations: subacute (*k* = 11), chronic (*k* = 13), mix-early (acute/subacute, *k* = 3), and mix-late (subacute/chronic, *k* = 6). Point estimates were larger in studies involving earlier-stage populations: mix-early [MD = 8.27, 95% CI (2.38, 14.16)], subacute [MD = 4.98, 95% CI (2.80, 7.15)], chronic [MD = 3.54, 95% CI (1.51, 5.57)], and mix-late [MD = 2.89, 95% CI (−4.79, 10.56)]. The test for subgroup differences was not statistically significant [*Q*-between(3) = 3.37, *P* = 0.34].

Regarding signal acquisition paradigms, no statistically significant difference was observed across subgroups [*P* = 0.62; *Q*-between(4) = 2.61]. MI was the most widely investigated strategy [*k* = 27, MD = 4.11, 95% CI (2.12, 6.09)]. AO included two studies [*k* = 2, MD = 5.97, 95% CI (−4.43, 16.37)]. MA included two studies [*k* = 2, MD = 9.34, 95% CI (0.09, 18.59)]. MI_AO and SSVEP were each represented by a single study (*k* = 1).

In terms of feedback modality, no statistically significant difference was observed across subgroups [*P* = 0.12; *Q*-between(2) = 4.21]. The largest point estimate was for FES [*k* = 12, MD = 6.21, 95% CI (3.19, 9.22)], followed by robotic device [*k* = 18, MD = 3.77, 95% CI (1.74, 5.80)] and visual feedback [*k* = 2, MD = 2.29, 95% CI (−12.28, 16.85)].

Comparator type was a significant source of between-group heterogeneity [*Q*-between(3) = 13.43, *P* < 0.01]. The pooled effect was largest against usual care or conventional rehabilitation [*k* = 12, MD = 6.70, 95% CI (4.43, 8.96)] and against device-assisted non-BCI controls [*k* = 5, MD = 4.48, 95% CI (0.31, 8.64)]. It was smaller against active dose-matched rehabilitation [*k* = 10, MD = 2.41, 95% CI (0.48, 4.34)] and against sham or neural-contingency controls [*k* = 6, MD = 1.97, 95% CI (−2.51, 6.45)], the latter not reaching statistical significance.

Effects did not differ significantly by study design [*Q*-between(1) = 2.31, *P* = 0.13]; point estimates were numerically larger in add-on trials [*k* = 10, MD = 5.93, 95% CI (3.45, 8.41)] than in comparative trials [*k* = 23, MD = 3.25, 95% CI (0.93, 5.57)].

Training dosage, examined in an exploratory meta-regression, could not be reliably modeled: dosage designs were narrowly clustered (71% of studies used five sessions per week), and the meta-regression explained essentially none of the variance in FMA-UE improvement (all *R*^2^ ≈ 0%). These analyses used study-level aggregate data and are subject to ecological bias. No dose-response signal was detectable within the reported range (Supplementary methods; Supplementary Table S6).

Several subgroups contained only two or three studies (motor attempt, action observation, and visual feedback), leaving these subgroup analyses underpowered. The absence of a statistically significant between-group difference should therefore be interpreted as a failure to detect a difference rather than as evidence of true equivalence.

### Publication bias assessment

**Figure 5:**
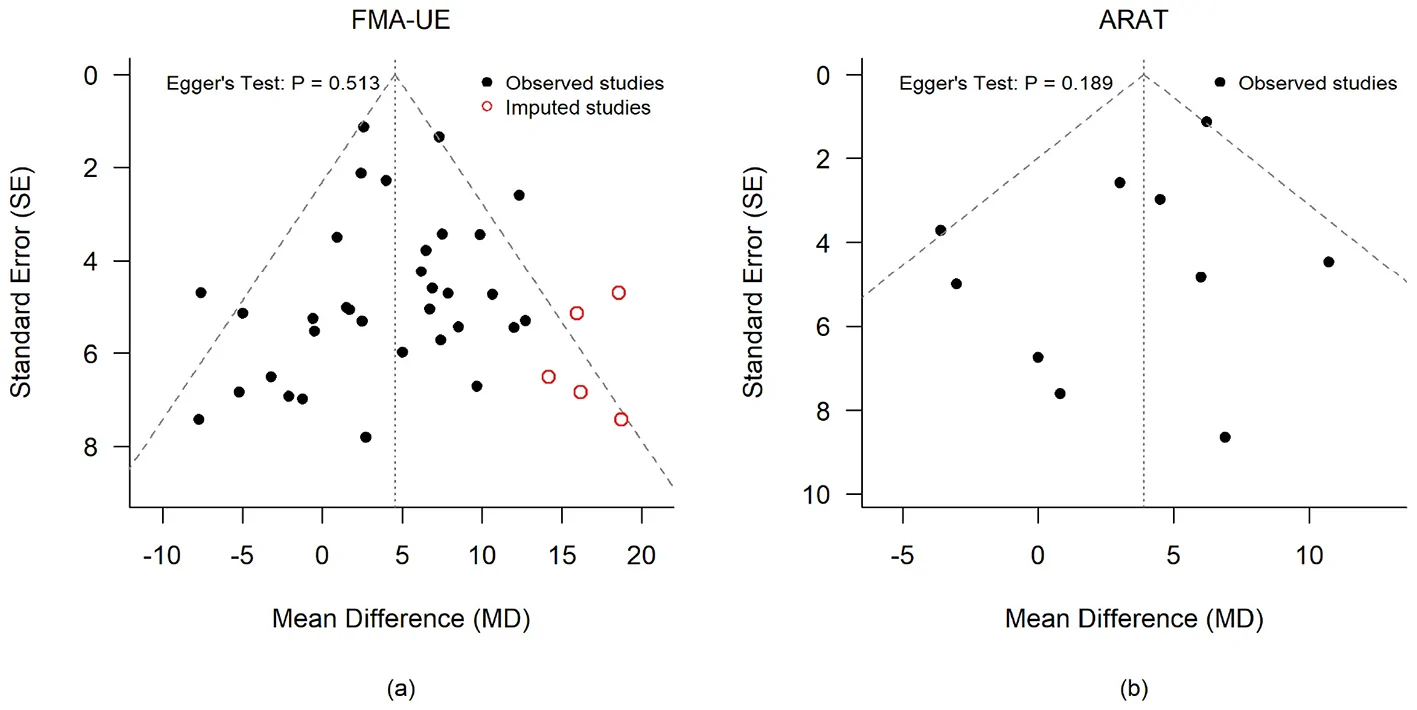
Funnel plot assessment for potential publication bias. **(a)** Funnel plot for the FMA-UE outcome, showing the observed studies (black circles) and studies imputed using the trim-and-fill method (red open circles). **(b)** Funnel plot for the ARAT outcome. FMA-UE, Fugl-Meyer Assessment-Upper Extremity; ARAT, Action Research Arm Test; SE, standard error.

For FMA-UE, visual inspection of the funnel plot (Figure 5a) indicated a broadly symmetrical distribution. Egger's linear regression test showed no significant asymmetry (*t* = −0.66, df = 31, *P* = 0.513). As an exploratory sensitivity analysis, the trim-and-fill method (L0 estimator) imputed five potentially missing studies, yielding an adjusted MD of 5.57 [95% CI (3.73, 7.41), *P* < 0.001]. The adjusted estimate was larger than the primary MD (4.55), which is atypical for trim-and-fill and likely reflects the L0 estimator placing imputed studies on the high-effect tail of the funnel, where fewer small studies were observed. The direction and statistical significance of the effect were preserved. For ARAT, the funnel plot appeared broadly symmetrical (Figure 5b), and Egger's test showed no significant asymmetry (*t* = −1.43, df = 8, *P* = 0.189). Because only five studies were available for WMFT, formal assessment of funnel plot asymmetry was not performed.

## Neuroplastic evidence underlying functional recovery: a qualitative synthesis

Given the methodological heterogeneity across included studies, a quantitative meta-analysis was not feasible for neurophysiological outcomes. Instead, we conducted a structured narrative synthesis for 22 studies reporting neuroplasticity-related outcomes, grouped broadly into three evidence streams: electrophysiological evidence (EEG/TMS; *k* = 11), hemodynamic or structural imaging evidence (fMRI/fNIRS/DTI; *k* = 8), and direct brain-behavior correlation analyses (*k* = 3). Three studies contributed to more than one stream. The synthesis below emphasizes recurring patterns while also noting that specific biomarkers and analytic pipelines varied considerably across studies. A detailed study-level summary of reported neuroplastic mechanisms and brain-behavior correlations is provided in Supplementary Tables S7, S8.

### Electrophysiological modulations

Studies using electrophysiological measures suggested that BCI training was associated with modulation of cortical excitability and task-related neural efficiency. Across EEG studies, a recurring pattern was enhanced or more focal ipsilesional sensorimotor rhythm modulation during motor tasks after intervention (27, 49). In some reports, this activity became more localized over the sensorimotor cortex in patients who showed better functional recovery (38, 40). However, the exact frequency-band profile and spatial distribution were not identical across studies, likely reflecting differences in task design, stroke stage, and analytic approach. In addition, one recent study reported altered prefrontal power ratios during BCI performance, raising the possibility that cognitive control and attentional allocation may also contribute to training-related effects (23).

Complementing these oscillatory changes, direct evidence from TMS showed that BCI training significantly increased the excitability of the corticospinal pathway. This was characterized by larger motor evoked potential (MEP) amplitudes and enhanced corticomuscular coherence (27), providing physiological validation that BCI-induced plasticity extends to the descending motor tracts.

### Hemodynamic and structural evidence

Hemodynamic and structural findings from fMRI, fNIRS, and DTI generally suggested that recovery after BCI training was accompanied by a reorganization of activation patterns rather than a uniform change in signal magnitude alone. Some fMRI studies described a shift from diffuse bilateral recruitment toward more lateralized activation in the ipsilesional hemisphere during motor tasks (35, 51). fNIRS studies further indicated that training-related changes could extend beyond primary sensorimotor regions, including altered activation in prefrontal areas that may reflect the engagement of broader task-control processes (24).

At the network level, several studies reported reorganization spanning local sensorimotor circuits and larger-scale functional systems. More local effects included strengthened connectivity within ipsilesional sensorimotor regions (36, 46) and changes involving subcortical-related motor circuits (41). More recent work also suggested that BCI-related recovery may be accompanied by altered connectivity in distributed networks, including attention-related and default mode systems (20). In addition, improved effective connectivity between premotor and motor regions has been reported (22), and preliminary DTI evidence suggested that these functional changes may be accompanied by structural remodeling within the ipsilesional corticospinal tract (21). Overall, these findings support a multi-level view of neuroplasticity, but the heterogeneity of modalities and metrics precludes a single unified model.

### Direct evidence of brain-behavior correlations

Quantitative associations between neurophysiological changes and clinical improvement were reported across multiple included studies, supporting a potential link between BCI-related plasticity and functional recovery. Detailed correlations between neural measures and functional outcomes are summarized in Supplementary Table S8.

In the electrophysiological domain, one study found a strong positive correlation between motor gains and increased ERD magnitude after training (*r* = 0.96) (27). In the hemodynamic domain, clinical improvement was associated with more lateralized or normalized fMRI activation patterns (*r* = 0.55) (51) and with the strength of connectivity in motor-related circuits (*r* ≈ 0.66–0.8) (41).

More recent studies also linked functional recovery to the engagement of broader control-related networks. Lu et al. (20) reported significant correlations between motor improvement and activation in attention-related (*r* = 0.791) and default mode networks (*r* = 0.762), while Ji et al. (24) found associations between ARAT scores and dorsolateral prefrontal cortex activation (*r* = 0.596–0.67). In addition, BCI decoding accuracy was associated with treatment gains in some studies (*r* = 0.526–0.71) (33, 43). These findings support the possibility that recovery depends not only on sensorimotor reorganization but also on the effective recruitment of cognitive-control processes. However, these associations remain study-specific and should not be interpreted as establishing a single causal pathway.

## Discussion

This systematic review quantifies the clinical efficacy of BCI-based post-stroke upper-limb rehabilitation and pairs it with a structured synthesis of neuroplasticity evidence from the same trials. The pooled analysis of 35 RCTs indicates that BCI training was associated with improved upper-limb motor function across three standard scales. The FMA-UE pooled estimate (MD = 4.55) reaches the lower bound of the clinically important difference for the FMA-UE (4.25–7.25 points), which Page et al. (54) established in chronic stroke with minimal-to-moderate upper-limb impairment. As our pooled sample spans a broader range of stroke stages and severities, we interpret this value as an approximate clinical reference rather than a validated threshold for the present population. Low-to-moderate heterogeneity for the primary outcome (*I*^2^ = 36.8%) indicates a consistent effect direction. These findings indicate that BCI benefits post-stroke upper-limb recovery, but this conclusion should be read cautiously, as most contributing trials were small and few were rated at low risk of bias. We examine the neurological outcomes reported in the included trials, which document the neural changes that accompany BCI training.

To identify potential sources of BCI treatment effects, we examined whether stroke stage, signal acquisition strategy, feedback modality, comparator type, study design and training dosage moderated FMA-UE improvement. Only comparator type emerged as a significant moderator: the estimated effect varied by the rigor of the control condition, with larger estimates against usual care and more modest estimates against sham-contingent controls. Importantly, the point estimate favored BCI in every comparator category, including the strictest sham-contingent controls. A modest difference against sham is expected on mechanistic grounds. In the included trials, sham-contingent controls generally held constant the elements BCI shares with active therapy: dose was matched, patients still attempted motor imagery, and peripheral input from electrical stimulation or a robotic device was comparable across arms. The two conditions differed mainly in whether feedback was locked to the patient's own neural activity. The sham comparison therefore isolates the incremental contribution of genuine neural contingency, a narrower quantity than the total benefit captured against less active controls (55). That this isolated estimate did not reach statistical significance indicates that, once shared elements such as dose, motor imagery engagement, and peripheral input are held constant, the independent contribution of neural contingency remains uncertain.

We conducted a narrative synthesis of the neurophysiological findings reported in the included trials. BCI training was accompanied by neural changes at multiple levels of organization. Whether these changes converge on a common direction across modalities cannot be determined from the current evidence base, given the small number of studies per modality and the absence of direct cross-modal comparisons. At the local level, TMS studies reported larger MEP amplitudes after BCI training, indicating increased corticospinal excitability (21, 27). At the regional level, EEG studies consistently reported stronger or more spatially focused modulation of the ipsilesional sensorimotor rhythm (27, 49). At the network level, fMRI and fNIRS studies found a shift from diffuse bilateral activation toward ipsilesional lateralization, accompanied by stronger functional connectivity within sensorimotor and frontoparietal circuits (20, 51). At the structural level, preliminary DTI evidence suggested remodeling within the ipsilesional corticospinal tract (21). These findings, each at a different organizational level and acquired with different measurement modalities, describe candidate neurophysiological changes associated with BCI training.

Beyond these sensorimotor changes, a smaller body of evidence also points to cognitive control networks. Across EEG, fNIRS, and fMRI studies, activation changes in attention-related networks, the default mode network, and the dorsolateral prefrontal cortex were linked to clinical improvement (20, 24, 35). This evidence is preliminary, as far fewer studies address this level than the sensorimotor one. The co-occurrence of sensorimotor and cognitive-control signals has also been noted in the motor-learning and stroke-recovery literature (56, 57). A defining feature of BCI is that it directly quantifies neural engagement, a dimension invisible in conventional rehabilitation. Across the included studies, higher decoding accuracy was associated with greater clinical benefit (33). This association is open to two interpretations. It may reflect better signal acquisition and decoding (58), or patients with stronger baseline cognitive control may both drive the BCI more accurately and respond better to training. Because signal quality depends on both technical and patient-level factors, the two cannot be disentangled with current data. Prospectively measuring cognitive control network function alongside decoding accuracy would help resolve this question.

The quantitative synthesis has several limitations. The pooled interventions varied in signal-acquisition paradigm and feedback modality and, in some studies, in accompanying co-interventions such as physiotherapy, transcranial direct current stimulation, or mindfulness practice, so the summary estimate should be read as an umbrella effect across a family of related but non-identical interventions rather than the effect of a single, uniform treatment, and the present findings therefore support the efficacy of BCI-based multimodal rehabilitation as a class rather than establishing the superiority of any specific configuration. Beyond this, several analyses were statistically constrained. Subgroup analyses for signal-acquisition strategy were substantially underpowered: motor attempt and action observation were represented by only two and three studies, respectively, and the non-significant between-group differences should be interpreted as an absence of detectable difference rather than as evidence of paradigm equivalence. The dose-response analyses were constrained by the narrow and highly clustered range of dosages across existing trials, which prevented meaningful modeling of sessions per week and left two of four predictors with *R*^2^ = 0%. The current evidence base therefore cannot address the dose-response question. The limited diversity of dosage protocols in existing trials is itself a key research gap, and future trials should deliberately vary training dosage to permit a proper test of dose-response. The WMFT estimate warrants particular caution. It rests on only five studies, so the observed heterogeneity (*I*^2^ = 68.1%) is itself imprecise and may be sensitive to individual trials. The FAS scores performance on functional motor tasks, an activity-level construct, whereas the FMA-UE assesses motor impairment. Activity-level performance is more influenced by compensation, task strategy, and context, which may make the WMFT more sensitive to differences in intervention protocol, stroke stage, and baseline severity across the five contributing trials. The WMFT result is therefore treated as preliminary. Risk of bias is a further concern, as only 8 of 35 studies were rated at low risk, and selective outcome reporting cannot be excluded in the smaller trials. Finally, the search was restricted to peer-reviewed articles and did not include gray literature, trial registries, or conference proceedings; in a technology-driven field where small pilot studies are common, this may increase the risk of publication bias, although Egger's test and trim-and-fill analysis did not indicate substantial small-study effects for the primary outcome.

The neuroplasticity synthesis carries distinct limitations. The 22 contributing studies differed too widely in imaging modality, analytic approach, and outcome metric to support quantitative pooling; the synthesis therefore captures directional patterns rather than precise effect sizes. All included studies used pre-post designs without formal mediation analysis, so the causal direction between neural change and functional recovery cannot be established from the available data.

## Conclusion

This systematic review found that BCI-based multimodal rehabilitation improves post-stroke upper-limb motor function, with the estimated effect varying by comparator type. Neuroplasticity outcomes were examined across electrophysiological, hemodynamic, and structural modalities in the included trials, but the current evidence is too heterogeneous and sparse to support a mechanistic account of how BCI training facilitates recovery. Further research is needed to determine whether and how training-associated neural changes relate causally to functional improvement.

## Data Availability

The original contributions presented in the study are included in the article/Supplementary material, further inquiries can be directed to the corresponding author.
