# Supplementary material for: Brain-computer interface for post-stroke upper-limb recovery: a systematic review integrating clinical efficacy with neuroplasticity evidence
Source: Front Neurol. 2026 Sep 3;17:1895484. doi: 10.3389/fneur.2026.1895484 (PMC13581672; doi:10.3389/fneur.2026.1895484)
Supplement: Supplementary file 1 [file Supplementary_file_1.docx]

**Supplementary Material**

**Supplementary Table S1**. PRISMA Checklist

| **Section and Topic** | **Item #** | **Checklist item** | **Location where item is reported** |
| --- | --- | --- | --- |
| **TITLE** | | |  |
| Title | 1 | Identify the report as a systematic review. | Title Page |
| **ABSTRACT** | | |  |
| Abstract | 2 | See the PRISMA 2020 for Abstracts checklist. | Abstract |
| **INTRODUCTION** | | |  |
| Rationale | 3 | Describe the rationale for the review in the context of existing knowledge. | **Introduction** |
| Objectives | 4 | Provide an explicit statement of the objective(s) or question(s) the review addresses. | Introduction |
| **METHODS** | | |  |
| Eligibility criteria | 5 | Specify the inclusion and exclusion criteria for the review and how studies were grouped for the syntheses. | Methods: Eligibility criteria and study selection |
| Information sources | 6 | Specify all databases, registers, websites, organisations, reference lists and other sources searched or consulted to identify studies. Specify the date when each source was last searched or consulted. | Methods: Search strategy |
| Search strategy | 7 | Present the full search strategies for all databases, registers and websites, including any filters and limits used. | Methods: Search strategy; Supplementary table 2 |
| Selection process | 8 | Specify the methods used to decide whether a study met the inclusion criteria of the review, including how many reviewers screened each record and each report retrieved, whether they worked independently, and if applicable, details of automation tools used in the process. | Methods: Eligibility criteria and study selection |
| Data collection process | 9 | Specify the methods used to collect data from reports, including how many reviewers collected data from each report, whether they worked independently, any processes for obtaining or confirming data from study investigators, and if applicable, details of automation tools used in the process. | Methods: Data extraction |
| Data items | 10a | List and define all outcomes for which data were sought. Specify whether all results that were compatible with each outcome domain in each study were sought (e.g. for all measures, time points, analyses), and if not, the methods used to decide which results to collect. | Methods: Data extraction; Table 1 |
|  | 10b | List and define all other variables for which data were sought (e.g. participant and intervention characteristics, funding sources). Describe any assumptions made about any missing or unclear information. | Methods: Data extraction |
| Study risk of bias assessment | 11 | Specify the methods used to assess risk of bias in the included studies, including details of the tool(s) used, how many reviewers assessed each study and whether they worked independently, and if applicable, details of automation tools used in the process. | Methods: Quality assessment |
| Effect measures | 12 | Specify for each outcome the effect measure(s) (e.g. risk ratio, mean difference) used in the synthesis or presentation of results. | Methods: Statistical analysis (Data synthesis) |
| Synthesis methods | 13a | Describe the processes used to decide which studies were eligible for each synthesis (e.g. tabulating the study intervention characteristics and comparing against the planned groups for each synthesis (item #5)). | Methods: Eligibility criteria and study selection |
|  | 13b | Describe any methods required to prepare the data for presentation or synthesis, such as handling of missing summary statistics, or data conversions. | Methods: Data extraction; Table 1 |
|  | 13c | Describe any methods used to tabulate or visually display results of individual studies and syntheses. | Methods: Statistical analysis; Results |
|  | 13d | Describe any methods used to synthesize results and provide a rationale for the choice(s). If meta-analysis was performed, describe the model(s), method(s) to identify the presence and extent of statistical heterogeneity, and software package(s) used. | Methods: Statistical analysis (Data synthesis) |
|  | 13e | Describe any methods used to explore possible causes of heterogeneity among study results (e.g. subgroup analysis, meta-regression). | Methods: Statistical analysis (Subgroup analysis) |
|  | 13f | Describe any sensitivity analyses conducted to assess robustness of the synthesized results. | Methods: Statistical analysis (Heterogeneity analysis and publication bias) |
| Reporting bias assessment | 14 | Describe any methods used to assess risk of bias due to missing results in a synthesis (arising from reporting biases). | Methods: Statistical analysis (Heterogeneity analysis and publication bias) |
| Certainty assessment | 15 | Describe any methods used to assess certainty (or confidence) in the body of evidence for an outcome. | Methods: Certainty of evidence assessment |
| **RESULTS** | | |  |
| Study selection | 16a | Describe the results of the search and selection process, from the number of records identified in the search to the number of studies included in the review, ideally using a flow diagram. | Results: Study selection and characteristics; Figure 1 |
|  | 16b | Cite studies that might appear to meet the inclusion criteria, but which were excluded, and explain why they were excluded. | Figure 1 |
| Study characteristics | 17 | Cite each included study and present its characteristics. | Results: Study selection and characteristics; Table 2 |
| Risk of bias in studies | 18 | Present assessments of risk of bias for each included study. | Results: Methodological quality and risk of bias; Figure 2 |
| Results of individual studies | 19 | For all outcomes, present, for each study: (a) summary statistics for each group (where appropriate) and (b) an effect estimate and its precision (e.g. confidence/credible interval), ideally using structured tables or plots. | Results: Effects on upper-limb functional outcomes; Figure 3 |
| Results of syntheses | 20a | For each synthesis, briefly summarise the characteristics and risk of bias among contributing studies. | Results: Study selection and characteristics; Methodological quality and risk of bias |
|  | 20b | Present results of all statistical syntheses conducted. If meta-analysis was done, present for each the summary estimate and its precision (e.g. confidence/credible interval) and measures of statistical heterogeneity. If comparing groups, describe the direction of the effect. | Results: Effects on upper-limb functional outcomes; Qualitative synthesis |
|  | 20c | Present results of all investigations of possible causes of heterogeneity among study results. | Results: Moderator analyses of FMA-UE; Dose-response relationship; Figure 4; Table 3 |
|  | 20d | Present results of all sensitivity analyses conducted to assess the robustness of the synthesized results. | Results: Publication bias assessment |
| Reporting biases | 21 | Present assessments of risk of bias due to missing results (arising from reporting biases) for each synthesis assessed. | Results: Publication bias assessment; Figure 5 |
| Certainty of evidence | 22 | Present assessments of certainty (or confidence) in the body of evidence for each outcome assessed. | Results: Certainty of evidence; Supplementary Table S4 |
| **DISCUSSION** | | |  |
| Discussion | 23a | Provide a general interpretation of the results in the context of other evidence. | Discussion |
|  | 23b | Discuss any limitations of the evidence included in the review. | Discussion: Study limitations |
|  | 23c | Discuss any limitations of the review processes used. | Discussion: Study limitations |
|  | 23d | Discuss implications of the results for practice, policy, and future research. | Discussion; Conclusions |
| **OTHER INFORMATION** | | |  |
| Registration and protocol | 24a | Provide registration information for the review, including register name and registration number, or state that the review was not registered. | Methods: Search strategy |
|  | 24b | Indicate where the review protocol can be accessed, or state that a protocol was not prepared. | Methods: Search strategy |
|  | 24c | Describe and explain any amendments to information provided at registration or in the protocol. | Methods: Protocol deviations |
| Support | 25 | Describe sources of financial or non-financial support for the review, and the role of the funders or sponsors in the review. | Funding |
| Competing interests | 26 | Declare any competing interests of review authors. | Conflicts of interest |
| Availability of data, code and other materials | 27 | Report which of the following are publicly available and where they can be found: template data collection forms; data extracted from included studies; data used for all analyses; analytic code; any other materials used in the review. | Data, material and/or code availability |

*From:*  Page MJ, McKenzie JE, Bossuyt PM, Boutron I, Hoffmann TC, Mulrow CD, et al. The PRISMA 2020 statement: an updated guideline for reporting systematic reviews. BMJ 2021;372:n71. doi: 10.1136/bmj.n71

For more information, visit: <http://www.prisma-statement.org/>

**Supplementary Table S2.** Search strategy

| Database | Step | Search algorithm |
| --- | --- | --- |
| Pubmed | 1 | “Stroke"[Mesh] OR stroke[Title/Abstract] OR poststroke[Title/Abstract] OR post-stroke[Title/Abstract] OR cerebrovascular accident*[Title/Abstract] OR cerebral infarction[Title/Abstract] OR cerebral hemorrhage[Title/Abstract] OR intracerebral hemorrhage[Title/Abstract]” |
|  | 2 | “Brain-Computer Interfaces"[Mesh] OR brain-computer interface*[Title/Abstract] OR brain computer interface*[Title/Abstract] OR BCI[Title/Abstract] OR brain-machine interface*[Title/Abstract] OR brain machine interface*[Title/Abstract] OR neural interface technolog*[Title/Abstract]” |
|  | 3 | #1 AND #2 |
| Web of science | #1 | TS=(("Strokes") OR ("Cerebrovascular Accident") OR ("Cerebrovascular Accidents") OR ("Cerebral Stroke") OR ("Cerebral Strokes") OR ("Stroke, Cerebral") OR ("Strokes, Cerebral") OR ("Cerebrovascular Apoplexy") OR ("Apoplexy, Cerebrovascular") OR ("Vascular Accident, Brain") OR ("Brain Vascular Accident") OR ("Brain Vascular Accidents") OR ("Vascular Accidents, Brain") OR ("Cerebrovascular Stroke") OR ("Cerebrovascular Strokes") OR ("Stroke, Cerebrovascular") OR ("Strokes, Cerebrovascular") OR ("Apoplexy") OR ("CVA (Cerebrovascular Accident)") OR ("CVAs (Cerebrovascular Accident)") OR ("Stroke, Acute") OR ("Acute Stroke") OR ("Acute Strokes") OR ("Strokes, Acute") OR ("Cerebrovascular Accident, Acute") OR ("Acute Cerebrovascular Accident") OR ("Acute Cerebrovascular Accidents") OR ("Cerebrovascular Accidents, Acute")) |
|  | #2 | (TS=(("Brain Computer Interfaces") OR ("Interface, Brain-Computer") OR ("Interfaces, Brain-Computer") OR ("Neural Interface Technology") OR ("Interface Technologies, Neural") OR ("Interface Technology, Neural") OR ("Neural Interface Technologies") OR ("Technologies, Neural Interface") OR ("Technology, Neural Interface") OR ("Brain-Computer Interface") OR ("Brain Computer Interface") OR ("Brain-Machine Interfaces") OR ("Brain-Machine Interface") OR ("Interface, Brain-Machine") OR ("Interfaces, Brain-Machine") OR ("Brain Machine Interface") OR ("Brain Machine Interfaces") OR ("Interface, Brain Machine") OR ("Interfaces, Brain Machine") OR ("Machine Interface, Brain") OR ("Machine Interfaces, Brain"))) |
|  | #3 | #1 AND #2 |
| Embase | 1 | ('strokes':ti,ab,kw OR 'cerebrovascular accident':ti,ab,kw OR 'cerebrovascular accidents':ti,ab,kw OR 'cerebral stroke':ti,ab,kw OR 'cerebral strokes':ti,ab,kw OR 'stroke, cerebral':ti,ab,kw OR 'strokes, cerebral':ti,ab,kw OR 'cerebrovascular apoplexy':ti,ab,kw OR 'apoplexy, cerebrovascular':ti,ab,kw OR 'vascular accident, brain':ti,ab,kw OR 'brain vascular accident':ti,ab,kw OR 'brain vascular accidents':ti,ab,kw OR 'vascular accidents, brain':ti,ab,kw OR 'cerebrovascular stroke':ti,ab,kw OR 'cerebrovascular strokes':ti,ab,kw OR 'stroke, cerebrovascular':ti,ab,kw OR 'strokes, cerebrovascular':ti,ab,kw OR 'apoplexy':ti,ab,kw OR 'cva (cerebrovascular accident)':ti,ab,kw OR 'cvas (cerebrovascular accident)':ti,ab,kw OR 'stroke, acute':ti,ab,kw OR 'acute stroke':ti,ab,kw OR 'acute strokes':ti,ab,kw OR 'strokes, acute':ti,ab,kw OR 'cerebrovascular accident, acute':ti,ab,kw OR 'acute cerebrovascular accident':ti,ab,kw OR 'acute cerebrovascular accidents':ti,ab,kw OR 'cerebrovascular accidents, acute':ti,ab,kw) AND ([embase]/lim OR [medline]/lim) |
|  | 2 | ('brain computer interfaces':ti,ab,kw OR 'interface, brain-computer':ti,ab,kw OR 'interfaces, brain-computer':ti,ab,kw OR 'neural interface technology':ti,ab,kw OR 'interface technologies, neural':ti,ab,kw OR 'interface technology, neural':ti,ab,kw OR 'neural interface technologies':ti,ab,kw OR 'technologies, neural interface':ti,ab,kw OR 'technology, neural interface':ti,ab,kw OR 'brain-computer interface':ti,ab,kw OR 'brain computer interface':ti,ab,kw OR 'brain-machine interfaces':ti,ab,kw OR 'brain-machine interface':ti,ab,kw OR 'interface, brain-machine':ti,ab,kw OR 'interfaces, brain-machine':ti,ab,kw OR 'brain machine interface':ti,ab,kw OR 'brain machine interfaces':ti,ab,kw OR 'interface, brain machine':ti,ab,kw OR 'interfaces, brain machine':ti,ab,kw OR 'machine interface, brain':ti,ab,kw OR 'machine interfaces, brain':ti,ab,kw) AND ([embase]/lim OR [medline]/lim) |
|  | 3 | 1 AND 2 |
| Scopus | 1 | ( TITLE-ABS-KEY ( ( "Strokes" ) OR ( "Cerebrovascular Accident" ) OR ( "Cerebrovascular Accidents" ) OR ( "Cerebral Stroke" ) OR ( "Cerebral Strokes" ) OR ( "Stroke, Cerebral" ) OR ( "Strokes, Cerebral" ) OR ( "Cerebrovascular Apoplexy" ) OR ( "Apoplexy, Cerebrovascular" ) OR ( "Vascular Accident, Brain" ) OR ( "Brain Vascular Accident" ) OR ( "Brain Vascular Accidents" ) OR ( "Vascular Accidents, Brain" ) OR ( "Cerebrovascular Stroke" ) OR ( "Cerebrovascular Strokes" ) OR ( "Stroke, Cerebrovascular" ) OR ( "Strokes, Cerebrovascular" ) OR ( "Apoplexy" ) OR ( "CVA (Cerebrovascular Accident)" ) OR ( "CVAs (Cerebrovascular Accident)" ) OR ( "Stroke, Acute" ) OR ( "Acute Stroke" ) OR ( "Acute Strokes" ) OR ( "Strokes, Acute" ) OR ( "Cerebrovascular Accident, Acute" ) OR ( "Acute Cerebrovascular Accident" ) OR ( "Acute Cerebrovascular Accidents" ) OR ( "Cerebrovascular Accidents, Acute" ) ) |
|  | 2 | (TITLE-ABS-KEY ( ( "Brain Computer Interfaces" ) OR ( "Interface, Brain-Computer" ) OR ( "Interfaces, Brain-Computer" ) OR ( "Neural Interface Technology" ) OR ( "Interface Technologies, Neural" ) OR ( "Interface Technology, Neural" ) OR ( "Neural Interface Technologies" ) OR ( "Technologies, Neural Interface" ) OR ( "Technology, Neural Interface" ) OR ( "Brain-Computer Interface" ) OR ( "Brain Computer Interface" ) OR ( "Brain-Machine Interfaces" ) OR ( "Brain-Machine Interface" ) OR ( "Interface, Brain-Machine" ) OR ( "Interfaces, Brain-Machine" ) OR ( "Brain Machine Interface" ) OR ( "Brain Machine Interfaces" ) OR ( "Interface, Brain Machine" ) OR ( "Interfaces, Brain Machine" ) OR ( "Machine Interface, Brain" ) OR ( "Machine Interfaces, Brain" ) ) |
|  | 3 | 1 AND 2 |
| CENTRAL | 1 | (("Strokes"):ti,ab,kw OR("Cerebrovascular Accident"):ti,ab,kw OR ("Cerebrovascular Accidents"):ti,ab,kw OR ("Cerebral Stroke"):ti,ab,kw OR ("Cerebral Strokes"):ti,ab,kw OR ("Stroke, Cerebral"):ti,ab,kw OR ("Strokes, Cerebral"):ti,ab,kw OR ("Cerebrovascular Apoplexy"):ti,ab,kw OR ("Apoplexy, Cerebrovascular"):ti,ab,kw OR ("Vascular Accident, Brain"):ti,ab,kw OR ("Brain Vascular Accident"):ti,ab,kw OR ("Brain Vascular Accidents"):ti,ab,kw OR ("Vascular Accidents, Brain"):ti,ab,kw OR ("Cerebrovascular Stroke"):ti,ab,kw OR ("Cerebrovascular Strokes"):ti,ab,kw OR ("Stroke, Cerebrovascular"):ti,ab,kw OR ("Strokes, Cerebrovascular"):ti,ab,kw OR ("Apoplexy"):ti,ab,kw OR ("CVA (Cerebrovascular Accident)"):ti,ab,kw OR ("CVAs (Cerebrovascular Accident)"):ti,ab,kw OR ("Stroke, Acute"):ti,ab,kw OR ("Acute Stroke"):ti,ab,kw OR ("Acute Strokes"):ti,ab,kw OR ("Strokes, Acute"):ti,ab,kw OR ("Cerebrovascular Accident, Acute"):ti,ab,kw OR ("Acute Cerebrovascular Accident"):ti,ab,kw OR ("Acute Cerebrovascular Accidents"):ti,ab,kw OR ("Cerebrovascular Accidents, Acute"):ti,ab,kw) |
|  | 2 | (("Brain Computer Interfaces"):ti,ab,kw OR ("Interface, Brain-Computer"):ti,ab,kw OR ("Interfaces, Brain-Computer"):ti,ab,kw OR ("Neural Interface Technology"):ti,ab,kw OR ("Interface Technologies, Neural"):ti,ab,kw OR ("Interface Technology, Neural"):ti,ab,kw OR ("Neural Interface Technologies"):ti,ab,kw OR ("Technologies, Neural Interface"):ti,ab,kw OR ("Technology, Neural Interface"):ti,ab,kw OR ("Brain-Computer Interface"):ti,ab,kw OR ("Brain Computer Interface"):ti,ab,kw OR ("Brain-Machine Interfaces"):ti,ab,kw OR ("Brain-Machine Interface"):ti,ab,kw OR ("Interface, Brain-Machine"):ti,ab,kw OR ("Interfaces, Brain-Machine"):ti,ab,kw OR ("Brain Machine Interface"):ti,ab,kw OR ("Brain Machine Interfaces"):ti,ab,kw OR ("Interface, Brain Machine"):ti,ab,kw OR ("Interfaces, Brain Machine"):ti,ab,kw OR ("Machine Interface, Brain"):ti,ab,kw OR ("Machine Interfaces, Brain"):ti,ab,kw) |
|  | 3 | 1 AND 2 |
| EBSCO | 1 | AB (("Strokes") OR ("Cerebrovascular Accident") OR ("Cerebrovascular Accidents") OR ("Cerebral Stroke") OR ("Cerebral Strokes") OR ("Stroke, Cerebral") OR ("Strokes, Cerebral") OR ("Cerebrovascular Apoplexy") OR ("Apoplexy, Cerebrovascular") OR ("Vascular Accident, Brain") OR ("Brain Vascular Accident") OR ("Brain Vascular Accidents") OR ("Vascular Accidents, Brain") OR ("Cerebrovascular Stroke") OR ("Cerebrovascular Strokes") OR ("Stroke, Cerebrovascular") OR ("Strokes, Cerebrovascular") OR ("Apoplexy") OR ("CVA (Cerebrovascular Accident)") OR ("CVAs (Cerebrovascular Accident)") OR ("Stroke, Acute") OR ("Acute Stroke") OR ("Acute Strokes") OR ("Strokes, Acute") OR ("Cerebrovascular Accident, Acute") OR ("Acute Cerebrovascular Accident") OR ("Acute Cerebrovascular Accidents") OR ("Cerebrovascular Accidents, Acute")) |
|  | 2 | AB (("Brain Computer Interfaces") OR ("Interface, Brain-Computer") OR ("Interfaces, Brain-Computer") OR ("Neural Interface Technology") OR ("Interface Technologies, Neural") OR ("Interface Technology, Neural") OR ("Neural Interface Technologies") OR ("Technologies, Neural Interface") OR ("Technology, Neural Interface") OR ("Brain-Computer Interface") OR ("Brain Computer Interface") OR ("Brain-Machine Interfaces") OR ("Brain-Machine Interface") OR ("Interface, Brain-Machine") OR ("Interfaces, Brain-Machine") OR ("Brain Machine Interface") OR ("Brain Machine Interfaces") OR ("Interface, Brain Machine") OR ("Interfaces, Brain Machine") OR ("Machine Interface, Brain") OR ("Machine Interfaces, Brain")) |
|  | 3 | 1 AND 2 |

**Supplementary Table S3.**RoB2 Scale

| Study | D1 | D2 | D3 | D4 | D5 | Overall |
| --- | --- | --- | --- | --- | --- | --- |
| Lu 2025 | Low | Some concerns | Low | Low | Low | Some concerns |
| Kim 2025 | Low | Low | Low | Low | Low | Low |
| He 2025 | Low | Low | Low | Low | Low | Low |
| Ji 2025 | Low | Some concerns | Low | Low | Low | Some concerns |
| Zhang J 2025 | Low | Some concerns | Low | Low | Low | Some concerns |
| Cantillo 2025 | Low | Low | Low | Low | Low | Low |
| Brunner 2024 | Low | Some concerns | Low | Low | Low | Some concerns |
| Zhang M 2025 | Low | Some concerns | Low | Low | Low | Some concerns |
| Krueger 2024 | Low | Low | Low | Low | Low | Low |
| Fu 2023 | Low | Some concerns | Low | Low | Low | Some concerns |
| Liao 2023 | Low | Some concerns | Low | Low | Low | Some concerns |
| Liu 2023 | Some concerns | Some concerns | Some concerns | High | Some concerns | High |
| Wang 2023 | Low | Some concerns | Low | Low | Low | Some concerns |
| Ma 2023 | Low | Some concerns | Low | Low | Low | Some concerns |
| Lee 2022 | Low | Some concerns | Low | Low | Low | Some concerns |
| Zanona 2022 | Low | Low | Low | Low | Low | Low |
| Guo 2022 | Some concerns | Some concerns | Some concerns | High | Some concerns | High |
| Li X 2022 | Low | Some concerns | Low | High | Some concerns | High |
| Zhan 2022 | Some concerns | Some concerns | Low | High | Some concerns | High |
| Hu 2021 | Some concerns | Some concerns | Low | Low | Some concerns | Some concerns |
| Chen 2021 | Low | Some concerns | Low | Low | Low | Some concerns |
| Miao 2020 | Some concerns | Some concerns | Low | High | Some concerns | High |
| Cheng 2020 | Low | Some concerns | Some concerns | High | Some concerns | High |
| Wu 2020 | Low | Some concerns | Low | Low | Low | Some concerns |
| Chen 2020 | Some concerns | Some concerns | Low | High | Some concerns | High |
| Biasiucci 2018 | Low | Low | Low | Low | Low | Low |
| Frolov 2017 | Low | Some concerns | Low | Low | Low | Some concerns |
| Jang 2016 | Low | Some concerns | Low | Low | Low | Some concerns |
| Kim 2016 | Low | Some concerns | Low | Low | Low | Some concerns |
| Pichiorri 2015 | Low | Some concerns | Low | Low | Low | Some concerns |
| Li M 2014 | Low | Some concerns | Low | High | Some concerns | High |
| Ang 2014a | Low | Some concerns | Low | Low | Low | Some concerns |
| Ang 2014b | Low | Some concerns | Low | Low | Low | Some concerns |
| Mihara 2013 | Low | Low | Low | Low | Low | Low |
| Ramos 2013 | Low | Low | Low | Low | Low | Low |

D1: Randomization process; D2: Deviations from intended interventions; D3: Missing outcome data; D4: Measurement of the outcome; D5: Selection of the reported result.

**Supplementary Figure S1.** Detailed forest plot of the FMA-UE.

**Supplementary Figure S2.** Detailed forest plot of the ARAT.

**Supplementary Figure S3.** Detailed forest plot of the WMFT.

**Subgroup analysis 1: based on BCI signal type**

**Supplementary Figure S4.** Detailed subgroup analysis by BCI signal strategy.

**Subgroup analysis 2: based on stroke stage**

**Supplementary Figure S5.** Detailed subgroup analysis by stroke chronicity..

**Subgroup analysis 3: based on BCI output type**

**Supplementary Figure S6.** Detailed subgroup analysis by BCI feedback modality.

**Supplementary Figure S7.** Subgroup analysis of BCI efficacy on FMA-UE by comparator type.

Notes: Studies are grouped into four comparator categories: usual care or conventional rehabilitation, device-assisted non-BCI controls, active dose-matched rehabilitation, and sham or neural-contingency controls. Pooled mean differences (random-effects, DerSimonian–Laird) are shown as diamonds for each subgroup. The test for subgroup differences was significant (Q-between(3) = 13.43, P < 0.01). Abbreviations: BCI, brain-computer interface; CI, confidence interval; FMA-UE, Fugl-Meyer Assessment–Upper Extremity; MD, mean difference.

**Supplementary Figure S8.** Subgroup analysis of BCI efficacy on FMA-UE improvement by study design (add-on versus comparative trials).

Notes: Forest plot of mean differences (MD) in FMA-UE improvement between BCI and control groups, stratified by study design. Add-on trials added BCI to a background therapy delivered to both arms, whereas comparative trials tested BCI against a different therapy. BCI, brain-computer interface; CI, confidence interval; FMA-UE, Fugl-Meyer Assessment-Upper Extremity; MD, mean difference.

**Supplementary Table S4.** Definition of pooled outcome measures

| Outcome | Full name | Construct | Score range | Direction | Metric pooled |
| --- | --- | --- | --- | --- | --- |
| FMA-UE | Fugl-Meyer Assessment, Upper Extremity motor subscale | Motor impairment | 0–66 | Higher = better (less impairment) | Mean difference |
| ARAT | Action Research Arm Test | Activity / functional capacity | 0–57 | Higher = better | Mean difference |
| WMFT–FAS | Wolf Motor Function Test, Functional Ability Scale | Functional movement quality | 0–75 (15 tasks × 0–5) | Higher = better | Mean difference |

Note: All three outcomes were pooled as mean differences because each scale was reported in consistent units and direction across the contributing studies. For the WMFT, all five studies used the Functional Ability Scale (FAS); no study contributed timed WMFT data.

**Supplementary Table S5.** Summary of Findings (GRADE)

| Outcomes | No. of participants (studies) | Effect estimate, MD (95% CI) | Certainty of the evidence (GRADE) | Anticipated absolute effects |
| --- | --- | --- | --- | --- |
| FMA-UE  Assessed with: Fugl-Meyer Assessment | 1,154  (33 RCTs) | MD 4.55  [2.84 to 6.25] | ⨁⨁⨁◯  MODERATE*^a^* | The intervention likely improves motor impairment. |
| ARAT  Assessed with: Action Research Arm Test | 311  (10 RCTs) | MD 3.90  [1.31 to 6.50] | ⨁⨁◯◯  LOW*^b,c^* | The intervention may improve dexterous function. |
| WMFT  Assessed with: Wolf Motor Function Test | 176  (5 RCTs) | MD 8.53  [3.01 to 14.04] | ⨁⨁◯◯  LOW*^b,c^* | The intervention may improve gross motor function. |

**Supplementary Methods.** Dose-response meta-regression

Restricted cubic spline meta-regression was applied to explore potential non-linear relationships between training dosage and FMA-UE improvement. Three knots were used, positioned at the 10th, 50th, and 90th percentiles of each dosage variable's observed distribution. The dosage variables examined were total training sessions, total training duration (weeks), sessions per week, and session duration (minutes). Studies contributed to each predictor analysis only if they reported the relevant dosage variable; a minimum of eight studies per predictor was required for restricted cubic spline estimation. Non-linearity was evaluated by fitting spline terms and testing overall model fit against the null using the omnibus QM statistic. Because this analysis was exploratory and the number of available studies per dosage parameter was limited, this flexible model was selected to assess whether any obvious non-linear pattern was present rather than assuming a strictly linear relationship a priori. When distributional constraints precluded three distinct knot positions (two or more knots coinciding owing to limited predictor variation), simple linear meta-regression was substituted for that predictor and reported accordingly. For analyses requiring a standardized metric (dose-response curve display), standardized mean differences were used. The meta-regression used study-level aggregate data only and is therefore susceptible to ecological bias.

**Supplementary Table S6.** Meta-regression analyses of the dose-response relationship for the FMA-UE outcome

| **Variable** | **k** | **Q_M_** | **Coefficient (beta) [95% CI]** | **P value** | **R^2^** |
| --- | --- | --- | --- | --- | --- |
| Total sessions | 31 | 0.17 | Non-linear fit (RCS model) | 0.92 | 0% |
| Total training duration (weeks) | 31 | 0.08 | Non-linear fit (RCS model) | 0.96 | 0% |
| Sessions per week | 31 | 1.54 | 0.11 [-0.07, 0.29] | 0.22 | 3.8% |
| Session duration (min) | 25 | 2.54 | Non-linear fit (RCS model) | 0.28 | 4.5% |

**Abbreviations:** FMA-UE, Fugl-Meyer Assessment-Upper Extremity; CI, confidence interval; RCS, restricted cubic spline.

**Supplementary Table S7.** Summary of Neuroplastic Mechanisms Reported in Included Studies

| Study ID | Measurement Tool | Neural Metric | Key Findings (Region & Direction) | Significance |
| --- | --- | --- | --- | --- |
| Lu 2025 | fMRI | BOLD Activation | Increased activation in High-Order Networks (Default Mode, Dorsal/Ventral Attention) | pFDR<0.05 |
|  | fMRI | Effective Connectivity | Enhanced information flow: Lesioned Motor Cortex → Transmodal Networks → Intact Motor Cortex | p<0.05 |
| Ji 2025 | fNIRS | HbO Concentration | Increased activation in bilateral Sensorimotor Cortex (SMC) & Medial Prefrontal Cortex (MPFC) | p<0.05 |
| Zhang J 2025 | fNIRS | Network Efficiency | Increased global brain network efficiency post-intervention. | p<0.05 |
| Ma 2023 | fMRI (Task) | BOLD Activation | Increased activation in ipsilesional Superior & Middle Frontal Gyrus and widespread motor areas. | p<0.01 |
| He 2025 | EEG | Power Ratios (DAR/DABR) | Decreased ratios in Prefrontal Cortex (indicating higher cognitive engagement) | p<0.01 |
|  | fNIRS | Functional Connectivity | Enhanced FC between Prefrontal Cortex, SMA, and M1 | p<0.05 |
| Kim 2025 | EEG | Effective Connectivity (PDC) | Enhanced connectivity from contralesional premotor to ipsilesional motor area | p=0.016 |
| Cantillo 2025 | DTI | Fractional Anisotropy (rFA) | Increased structural integrity trend in ipsilesional Corticospinal Tract | Trend (n.s.) |
|  | TMS | Corticospinal Excitability | Increased MEP amplitude in ipsilesional hemisphere | Observed |
| Krueger 2024 | EEG / TMS | ERD / MEP | Enhanced Beta-band ERD and increased corticospinal excitability | p<0.05 |
| Ma 2023 | fMRI | Graph Theory (Topology) | Optimized topology in Visual & Dorsal Attention Networks | p<0.05 |
| Liao 2023 | EEG | ERD Topography | Focalized activation in Sensorimotor Cortex | Qualitative |
| Lee 2022 | EEG | Power Spectrum | Increased Beta power; Decreased Alpha power in frontal/central areas | p<0.05 |
| Zhan 2022 | EEG | Node strength | Increased Node Strength in the Contralesional Sensorimotor Cortex (C4). | P=0.035 |
| Wu 2020 | fMRI | Functional Connectivity | Increased inter-hemispheric connectivity (Premotor - Parietal) | p=0.003 |
| Chen 2020 | EEG | ERD | Enhanced ERD in ipsilesional Sensorimotor Cortex | p=0.032 |
| Biasiucci 2018 | EEG | Functional Connectivity | Increased connectivity within ipsilesional motor cortex (Beta band) | p=0.011 |
| Pichiorri 2015 | EEG | ERD & Connectivity | Stronger desynchronization & Increased intra-hemispheric connectivity | p<0.05 |
| Li 2014 | EEG | ERD | Increased ERD in the affected Sensorimotor Cortex (SMC). | p<0.05 |
| Mihara 2013 | NIRS | HbO Activation | Increased activation in ipsilesional Premotor Area | p<0.001 |
| Ramos 2013 | fMRI | Laterality Index (LI) | Shift towards ipsilesional activation ("Normalization") | p=0.02 |

Abbreviations: BOLD: Blood-Oxygen-Level-Dependent; DTI: Diffusion Tensor Imaging; PDC: Partial Directed Coherence; HbO: Oxygenated Hemoglobin; DAR: Delta/Alpha Ratio; DABR: Delta/Alpha+Beta Ratio; MEP: Motor Evoked Potential; ERD: Event-Related Desynchronization; PFC: Prefrontal Cortex; SMA: Supplementary Motor Area; PMC: Premotor Cortex; DMN: Default Mode Network.

**Supplementary Table S8.** Summary of Brain-Behavior Correlations in Included RCTs

| Study ID | Functional Outcome | Neural Metric | Brain Region / Network | Correlation | Significance |
| --- | --- | --- | --- | --- | --- |
| Lu 2025 | sEMG improvement | ↑ BOLD Activation | Ventral Attention Network | r=0.791 | p<0.001 |
|  | sEMG improvement | ↑ BOLD Activation | Default Mode Network | r=0.762 | p<0.001 |
|  | sEMG improvement | ↑ BOLD Activation | Fronto-Parietal Network | r=0.722 | p<0.001 |
|  | sEMG improvement | ↑ BOLD Activation | Ipsilesional Motor Cortex | r=0.589 | p=0.006 |
| Ji 2025 | ARAT improvement | ↑ HbO (Activation) | Right DLPFC (Prefrontal) | r=0.671 | p=0.003 |
|  | ARAT improvement | ↑ HbO (Activation) | Left DLPFC (Prefrontal) | r=0.592 | p=0.012 |
| Kim 2025 | MRC-WE (Strength) | ↑ Effective Connectivity | Ipsilesional Premotor → Motor | r=0.608 | p=0.027 |
|  | FMA-Distal | ↑ Effective Connectivity | Contralesional → Ipsilesional | r=0.568 | p=0.043 |
| Zhang J 2025 | FMA / MBI gains | ↑ Network Efficiency | Global Brain Network | r≈0.35 | p<0.05 |
| He 2025 | FMA-UE gains | ↓ EEG Power Ratios | Prefrontal Cortex (Fp1) | Negative | p<0.05 |
| Cantillo 2025 | FMA-UE gains | ↑ Structural Integrity (rFA) | Ipsilesional Corticospinal Tract | Positive | Trend (n.s.) |
| Krueger 2024 | FMA-UE score | ↑ Beta ERD Strength | Ipsilesional Motor Cortex | r=0.96 | p=0.044 |
| Ma 2023 | FMA-UE improvement | ↑ Network Assortativity | Dorsal Attention Network | r=0.498 | p=0.011 |
| Liu 2023 | FMA-UE improvement | ↑ Efficiency (Reaction Time) | Alert / Executive Networks | r≈0.40 | p<0.01 |
| Guo 2022 | FMA improvement | ↑ BCI Accuracy | Occipital Cortex (Visual) | r=0.714 | p=0.032 |
| Wu 2020 | WMFT score | ↑ Functional Connectivity | Premotor - Parietal - Putamen | r=0.814 | p<0.001 |
|  | ARAT score | ↑ Functional Connectivity | Premotor - Parietal - Putamen | r=0.701 | p=0.005 |
| Biasiucci 2018 | FMA-UE improvement | ↑ Functional Connectivity | Ipsilesional Sensorimotor (Beta) | r=0.48 | p<0.001 |
| Frolov 2017 | ARAT improvement | ↑ BCI Accuracy | Sensorimotor Cortex | r=0.52 | p=0.002 |
| Pichiorri 2015 | FMA effectiveness | ↑ Intra-hemispheric FC | Ipsilesional Hemisphere | r=0.604 | p=0.024 |
| Li M 2014 | FMA score | ↑ ERD Strength | Ipsilesional Sensorimotor | r=−0.546 | p=0.003 |
| Mihara 2013 | FMA hand/finger | ↑ HbO Activation | Ipsilesional Premotor Area | r=0.61 | p<0.005 |
| Ramos 2013 | FMA improvement | ↑ fMRI Laterality Index | Motor & Premotor Cortex | r=0.55 | p=0.05 |
| Ang 2014a | FMA improvement | ↓ rBSI (Improved Symmetry) | Whole Brain EEG | r=−0.616 | p=0.044 |
